# Surgical Management of Non-Metastatic Pancreatic Cancer in the United Kingdom: Results of a Nationwide Survey on Current Practice

**DOI:** 10.3389/fonc.2021.791946

**Published:** 2021-12-23

**Authors:** Georgios Gemenetzis, Siobhan McKay, Samir Pathak, John Moir, Richard Laing, Nigel B. Jamieson, Alastair L. Young, Nikolaos A. Chatzizacharias, Francesco Giovinazzo, Keith J. Roberts

**Affiliations:** ^1^ Department of Hepatobiliary (HPB) and Transplant Surgery, Royal Infirmary Edinburgh, Edinburgh, United Kingdom; ^2^ Liver Unit, Queen Elizabeth University Hospital Birmingham, Birmingham, United Kingdom; ^3^ Department of Surgery, University Hospitals Bristol NHS Foundation Trust, Bristol, United Kingdom; ^4^ Department of Hepatobiliary, Pancreatic and Transplant Surgery, The Freeman Hospital, Newcastle, United Kingdom; ^5^ West of Scotland Pancreatic Unit, Glasgow Royal Infirmary, Glasgow, United Kingdom; ^6^ Department of Pancreatic Surgery, St James’s University Hospital, Leeds, United Kingdom; ^7^ Department of General Surgery and Liver Transplantation, Fondazione Policlinico Universitario Agostino Gemelli IRCCS, Roma, Italy

**Keywords:** pancreatic cancer, survey, neoadjuvant, surgery, practice

## Abstract

**Background:**

It is presently unclear what clinical pathways are followed for patients with non-metastatic PDAC in specialised centres for pancreatic surgery across the United Kingdom (UK).

**Methods:**

Between August 2019 and August 2020 an electronic survey was conducted aiming at a national cohort of pancreatic surgeons in the UK. Participants replied to a list of standardised questions and clinical vignettes, and data were collected and analysed focusing on management preferences, resectability criteria, and contraindications to surgery.

**Results:**

Within the study period, 65 pancreatic surgeons from 27 specialist centres in the UK (96%) completed the survey. Multidisciplinary team meetings are utilised universally for the management of patients with PDAC, however, different staging systems for resectability classification are being applied. In borderline resectable PDAC, most surgeons were keen to proceed with surgical exploration post NAT, but differences were noted in preferred chemotherapy regimens. Surgeons from standard volume institutions performed fewer vein resections annually and were more likely to deem patients with locally advanced PDAC as unresectable. Intra-institutional variability in patient management was also present and ranging between 20-80%.

**Conclusions:**

Significant variability in the surgical management of non-metastatic PDAC was identified both on inter- and intra-institutional level.

## Introduction

Pancreatic adenocarcinoma (PDAC) remains a devastating disease with an extremely poor prognosis (1). The 5-year overall survival (OS) rate in the United Kingdom (UK) is approximately 7% and has not improved significantly in the past 30 years (2). Furthermore, there is a 17% increase in the incidence of PDAC cases at the same time period (3) resulting to more than 10,000 new diagnoses annually in the UK. In patients with non-metastatic PDAC, margin-negative (R0) surgical resection of the primary tumour is the primary therapeutic approach to achieve long-term survival (4, 5). Advancements in surgical quality and perioperative care and the introduction of more efficient perioperative systemic treatment have further improved patient outcomes (6, 7).

The paradigm of surgical management is shifting in patients with borderline resectable (BR) and locally advanced (LA) PDAC. Several classification systems in the literature define BR and LA-PDAC (8–10) focusing on variable degrees of vessel involvement by the tumour; yet the presence of different staging systems results in evolving definitions and a nuanced distinction between them (11). In BR-PDAC, the introduction of neoadjuvant/induction systemic treatment (NAT) was based on its potential advantages: addressing occult micrometastatic disease in the preoperative setting, avoiding unnecessary surgery in tumours with aggressive biology, increasing the likelihood of R0 resection, and improving delivery rates of systemic treatment. Recent prospective trials have demonstrated a potential benefit in overall survival in patients with BR-PDAC who underwent NAT (12, 13), and emerging data suggest potential future practice changes.

In the UK, the National Institute for Health and Care Excellence (NICE) pancreatic cancer guidelines recommend surgery for resectable cancer and advise the utilisation of NAT in resectable and BR-PDAC only as part of clinical trials (14). However, trial availability varies over time and patient recruitment can often be challenging. Therefore, it appears to be presently unclear what treatment pathways are chosen by the various specialist pancreatic surgical teams within the UK. A previous prospective audit on diagnostic pathways in PDAC (RICOCHET) demonstrated wide variations in practice and indicated the need for clinical pathway optimisation (15). The present study aims to further characterize variations in surgical management of patients with non-metastatic PDAC in specialised centres across the UK, assess and investigate differences in practice, and determine potential parameters for pathway standardisation.

## Methods

### Study Design and Participants

This study is a nationwide electronic survey that was carried out between August 2019 and August 2020. The survey was constructed on a web-based platform [REDCap, Vanderbilt University, TN, USA (16)] and was distributed *via* email, following established guidelines on electronic surveys (17). It was designed to target all consultant surgeons who hold a permanent or locum post and perform pancreatic surgery in institutions within the National Health System (NHS) across the UK. Identification of potential participants was performed through direct communications with every specialist unit in the UK. Senior trainees and fellows were excluded from this study. Eligible surgeons received an official email with the survey details and reminder emails were sent to participants who did not respond during the course of the study. Participants were informed in advance that the survey would require approximately 10-15 minutes to complete and that they needed to provide personal identification details including name, email address, and hospital. Additional information was asked to be provided regarding the annual volume of pancreatic resections performed in the institution of each participant. No rewards were offered for participation in the survey.

### Survey Contents: Questions and Clinical Vignettes

The survey was designed to assess surgical practice in PDAC focusing mainly on anatomical considerations, surgical techniques, and application of perioperative systemic treatment. An additional focus of the study was to acquire information regarding the organisation of institutional multidisciplinary team (MDT) meetings and structure of patient pathways. The rationale was to explore in detail differences in individual practices regarding the management of patients with non-metastatic PDAC. Therefore, the survey questionnaire was divided into two sections: the first included questions regarding practice characteristics and surgical decision-making, and the second comprised of five clinical vignettes. Most of the individual questions allowed one answer, however, select ones prompted participants to check multiple answers that applied.

The vignettes corresponded to five clinical scenarios that were created based on real patients (**Supplementary File 1**). Four vignettes included cases that covered the range of non-metastatic PDAC management (resectable, borderline resectable, and locally advanced tumours) and the fifth vignette addressed the management of a patient with a resectable pancreatic head primary and an indeterminate lung lesion. The vignette description contained all pertinent information to the participants, including patient demographics, clinical details, and anatomical characteristics of the tumour – de-identified CT images of real patients were utilised in three scenarios for clarity. In vignettes with BR-PDAC and LA-PDAC, all patients had previously received NAT with variable degrees of radiological and biomarker response. Three of the presented cases included follow-up questions to better assess surgical rationale. The full survey is available in the appendix (**Supplementary File 1**).

### Statistical Analysis

For optimal identification of surgical management, participants were stratified in three tiers, based on the volume of pancreatic resections in their institutions. Since all participants are practising in dedicated hepatopancreatobiliary centres identified as high-volume in the literature [>20 pancreatectomies annually (18)], the stratification occurred as follows: standard volume (<50 pancreatectomies/year), high volume (50-100 pancreatectomies/year), and very high volume (>100 pancreatectomies/year).

All participant data were de-identified prior to final analysis. Categorical and non-parametric data are presented as numbers and percentages. Continuous variables were compared with the Mann-Whitney *U* test and categorical variables with the *χ^2^* test. Intra-institutional variability in replies was calculated using Krippendorf’s α co-efficient on nominal data, and results were interpreted and presented as percentages (0-100%). For all tests, statistical significance was accepted with a two-sided *p* value of <0.05. The SPSS statistical software version 25.0 (SPSS Inc., Chicago, IL) was utilised for statistical analysis.

## Results

Within the study period, approximately 120 individual emails were sent to candidate participants, excluding reminder communications. In total, 65 eligible participants completed the survey, accounting approximately for 58% of all estimated HPB consultants in the UK and representing 27 of the 28 national referral centres. The median completion rate of the survey was 94.6% and all responses were included in the final analysis. The majority of the participants practise in academic institutions (n= 55) and 21 surgeons are also performing liver and/or pancreas transplantation. Regarding geographical distribution, 72% of the responders were based in England (n=47), 23% in Scotland (n=15), 3% in Wales (n=2), and 2% in Northern Ireland (n=1). According to the predetermined volume-based stratification, 14 surgeons practise in standard-volume (22%), 20 in high-volume (31%), and 31 in very high-volume institutions (48%).

All participants stated that MDT meetings are conducted in their respective institutions for evaluation and management of PDAC patients (**Table 1**). Most MDT meetings utilise the National Comprehensive Cancer Network (NCCN) guidelines for assessment of tumour resectability (19) (69%); surgeons from very high-volume centres reported increased adherence to the NCCN guidelines compared to standard-volume institutions (*p*=0.036). Interestingly, 14% of participants replied that they do not utilise a dedicated resectability classification system. Additionally, in the vast majority of cases MDT reports are presented as free text and only 3% of participants mentioned outcome reporting in a pre-drafted template. Most surgeons find useful the application of a universal resectability classification system nationally (62%) and approximately half of them support the standardisation of MDT reporting across the UK.

**Table 1:** Surgeon preferences for management of non-metastatic pancreatic cancer stratified by annual institutional volume of pancreatic resections.

Management Preferences	All (n = 65)	Standard volume (n = 14)	High volume (n = 20)	Very high volume (n = 31)	p-value
NAT in resectable PDAC, *n* (%)					0.106
Never	42 (65%)	12 (86%)	11 (55%)	19 (61%)	
Selectively	20 (31%)	2 (14%)	6 (30%)	12 (39%)	
Routinely	3 (4%)	0 (0%)	3 (15%)	0 (0%)	
Rationale for NAT in resectable PDAC, *n* (%)					
Clinical trial	3 (5%)	1 (7%)	0 (0%)	2 (6%)	0.191
Increased risk for R1 resection	9 (14%)	1 (7%)	5 (25%)	3 (10%)	
Low PS	2 (3%)	0 (0%)	0 (0%)	2 (6%)	
Preoperative pancreatitis	1 (2%)	0 (0%)	0 (0%)	1 (3%)	
Vessel contact	4 (6%)	0 (0%)	1 (5%)	3 (10%)	
Offer resection in resectable patients with high CA 19-9, *n* (%)	56 (86%)	13 (93%)	16 (80%)	27 (87%)	0.289
CA 19-9 cut-off for NAT in resectable patients, (U/ml)^a^					
< 250	5 (8%)	2 (14%)	1 (5%)	2 (6%)	0.415
250-500	12 (18%)	2 (14%)	4 (20%)	6 (19%)	
500-1000	7 (11%)	2 (14%)	1 (5%)	4 (13%)	
>1000	31 (48%)	7 (50%)	9 (45%)	15 (48%)	
Staging system for resectability classification in MDT, *n* (%)					
NCCN	45 (69%)	7 (50%)	13 (65%)	25 (81%)	**0.036**
Alliance	3 (5%)	1 (7%)	1 (5%)	1 (3%)	
MD Anderson	4 (6%)	2 (14%)	1 (5%)	1 (3%)	
Other	2 (3%)	0 (0%)	1 (5%)	1 (3%)	
None	9 (14%)	4 (29%)	3 (15%)	2 (6%)	
Multiple	2 (3%)	0 (0%)	1 (5%)	1 (3%)	
Universal resectability classification system, *n* (%)					
Useful/very useful	40 (62%)	8 (57%)	13 (65%)	19 (61%)	0.654
Not useful	25 (38%)	6 (43%)	7 (35%)	12 (39%)	
MDT report format, *n* (%)					0.565
Free text description	63 (97%)	13 (93%)	20 (100%)	30 (97%)	
Template with options	2 (3%)	1 (7%)	0 (0%)	1 (3%)	
Standardisation of MDT reporting, *n* (%)					
Definitely/Yes	33 (51%)	8 (57%)	8 (40%)	17(55%)	0.339
Potentially	30 (46%)	6 (43%)	12 (60%)	12 (39%)	
No	2 (3%)	0 (0%)	0 (0%)	2 (6%)	

NAT, neoadjuvant treatment; PDAC, pancreatic adenocarcinoma; CA19-9, carcinoembryonic antigen; MDT, multidisciplinary tumour board; NCCN, National Comprehensive Cancer Network; ^**a**^missing values n=10 (15%). P-values in bold are statistically significant.

Surgeon preferences on the management of non-metastatic PDAC are available in **Tables 1**, **2**. For upfront resectable PDAC, 65% of participants would never offer NAT in patients with performance status of 0-1, even in the presence of high CA19-9 levels (86%). Surgeons who supported the utilisation of NAT in resectable PDAC would offer it in patients with increased risk of positive margin (R1) resection due to contact of the tumour with the portal/superior mesenteric complex (n=13), or within a clinical trial (n=3).

**Table 2:** Technical surgical considerations in the management of borderline resectable and locally advanced pancreatic adenocarcinoma.

Management Preferences	All (n = 65)	Standard volume (n = 14)	High volume (n = 20)	Very high volume (n = 31)	p-value
Vascular resections in past 2 years, *n* (%)^a^					**<0.001**
0-1	3 (5%)	0 (0%)	0 (0%)	3 (10%)	
2-5	29 (45%)	12 (86%)	7 (35%)	10 (32%)	
6-10	20 (31%)	1 (7%)	9 (45%)	10 (32%)	
>10	12 (18%)	1 (7%)	3 (15%)	8 (26%)	
Preferred technique for vascular reconstruction, *n* (%)					0.286
Venorrhaphy only	3 (5%)	1 (7%)	1 (5%)	2 (6%)	
End-to-end anastomosis only	9 (14%)	0 (0%)	0 (0%)	4 (13%)	
Interposition graft only	0 (0%)	0 (0%)	0 (0%)	0 (0%)	
Multiple based on individual patient	54 (83%)	11 (79%)	18 (90%)	25 (81%)	
Vein characteristics, *n* (%)^b^ Length of involvement					0.693
Important/very important	54 (83%)	13 (93%)	17 (85%)	24 (77%)	
Somewhat important	3 (5%)	0 (0%)	1 (5%)	2 (6%)	
Not important	3 (5%)	1 (7%)	1 (5%)	1 (3%)	
Degree of circumferential involvement					0.647
Important/very important	48 (74%)	11 (79%)	14 (70%)	23 (74%)	
Somewhat important	10 (15%)	2 (14%)	3 (15%)	5 (16%)	
Not important	2 (3%)	0 (0%)	1 (5%)	1 (3%)	
Presence of narrowing					0.161
Important/very important	41 (63%)	11 (79%)	9 (45%)	21 (68%)	
Somewhat important	15 (23%)	2 (14%)	6 (30%)	7 (23%)	
Not important	5 (8%)	0 (0%)	4 (20%)	1 (3%)	
Cavernous transformation					0.326
Important/very important	59 (91%)	14 (100%)	18 (90%)	27 (87%)	
Somewhat important	1 (2%)	0 (0%)	0 (0%)	1 (3%)	
Not important	1 (2%)	0 (0%)	0 (0%)	1 (3%)	
1^st^ jejunal branch involvement					0.105
Important/very important	53 (82%)	14 (100%)	14 (70%)	25 (81%)	
Somewhat important	6 (9%)	0 (0%)	2 (10%)	4 (13%)	
Not important	3 (5%)	0 (0%)	1 (5%)	2 (6%)	
Artery characteristics, *n* (%)^c^ Length of involvement					0.628
Important/very important	57 (88%)	12 (86%)	18 (90%)	27 (87%)	
Somewhat important	2 (3%)	1 (7%)	0 (0%)	1 (3%)	
Not important	5 (8%)	0 (0%)	2 (10%)	3 (10%)	
Degree of circumferential involvement					0.342
Important/very important	62 (95%)	14 (100%)	19 (95%)	29 (94%)	
Somewhat important	1 (2%)	0 (0%)	1 (5%)	0 (0%)	
Not important	2 (3%)	0 (0%)	0 (0%)	2 (6%)	
Presence of narrowing					0.531
Important/very important	54 (83%)	12 (86%)	18 (90%)	24 (77%)	
Somewhat important	4 (6%)	2 (14%)	0 (0%)	2 (6%)	
Not important	7 (11%)	0 (0%)	2 (10%)	5 (16%)	

SMA, superior mesenteric artery; ^**a**^missing values n=1 (2%); ^**b**^missing values n=5 (8%); ^**c**^missing values n=1 (2%). P-values in bold are statistically significant.

**Figure 1:**
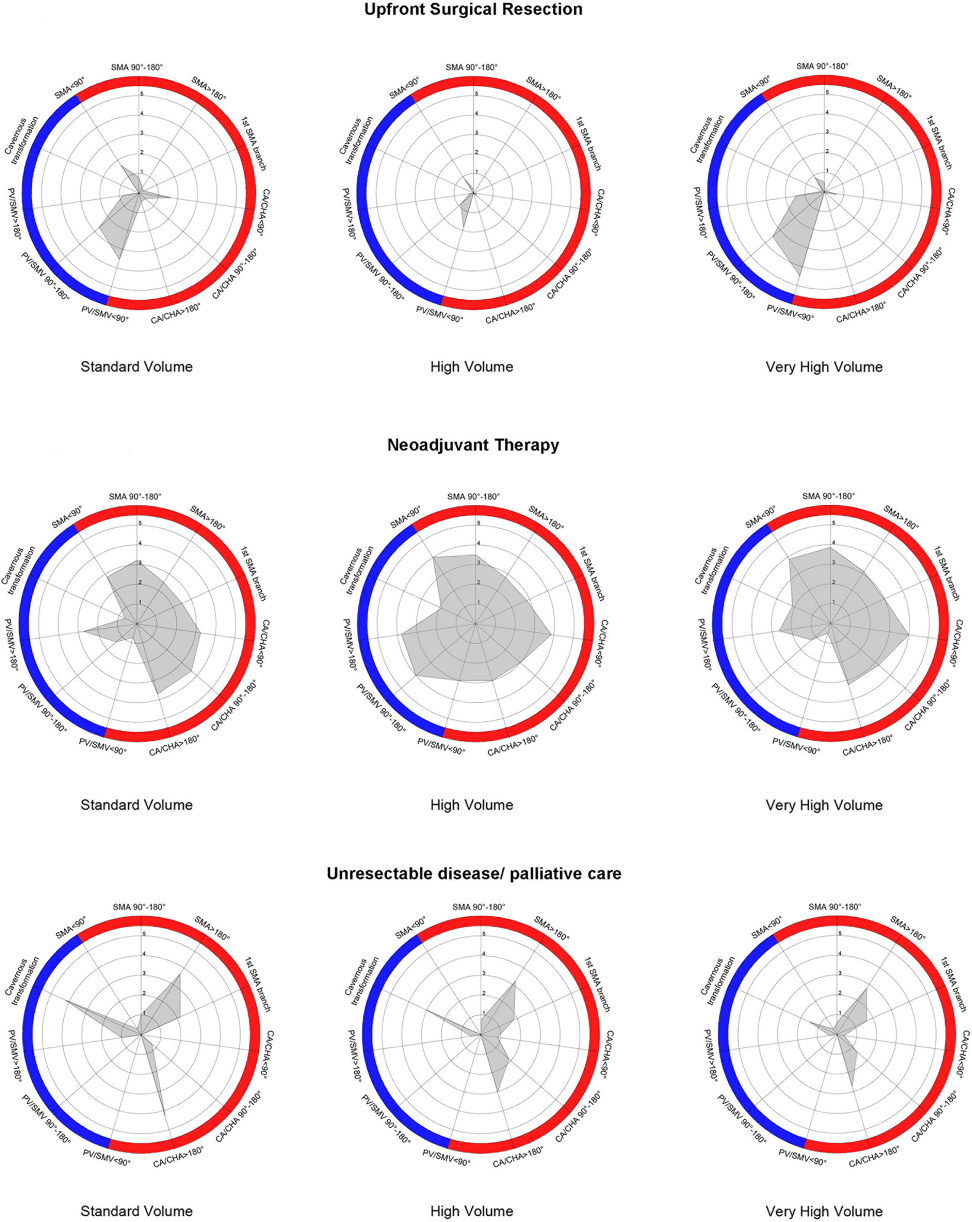
Radar chart depiction of upfront surgical resection, utilisation of neoadjuvant treatment, and declaration of unresectability in initial PDAC diagnosis based on variable vessel involvement by the primary tumour between the three volume-related tiers. Blue-coloured and red-coloured areas indicate different degrees of venous and arterial involvement, respectively; 1-5 enumeration refers to percentage of responses (1 = 20%, 5 = 100%).

Participants practising in very high-volume institutions for pancreatic surgery performed statistically significantly more vascular resections in pancreatectomies compared to ones in standard or high-volume centres (*p*<0.001). In BR and LA-PDAC, no differences were identified between surgeons regarding preferred technique for vascular reconstruction. Similarly, the importance of radiological involvement of regional vascular structures by the tumour was equally distributed: in veins length of involved segment and presence of cavernous transformation, and in arteries degree of circumferential involvement and presence of narrowing appear to be more significant in preoperative assessment (**Table 2**). **Figure 1** demonstrates the distribution of upfront surgical resection, NAT, and declaration of unresectability based on different degrees of venous and arterial involvement between participants in the three volume-based tiers (**Figure 1**; baseline histogram available in **Supplementary File 2**). Surgeons in high-volume centres were less likely to offer upfront resection in patients with tumours with any degree of vessel involvement compared to surgeons in the other two tiers. Additionally, surgeons in standard-volume institutions offered NAT in fewer cases and declared more patients unresectable, especially in the presence of cavernous transformation or encasement of the superior mesenteric artery (>180° involvement). Despite management variations, statistically significant differences were not identified between the three tiers.

### Clinical Vignettes

**Figure 2:**
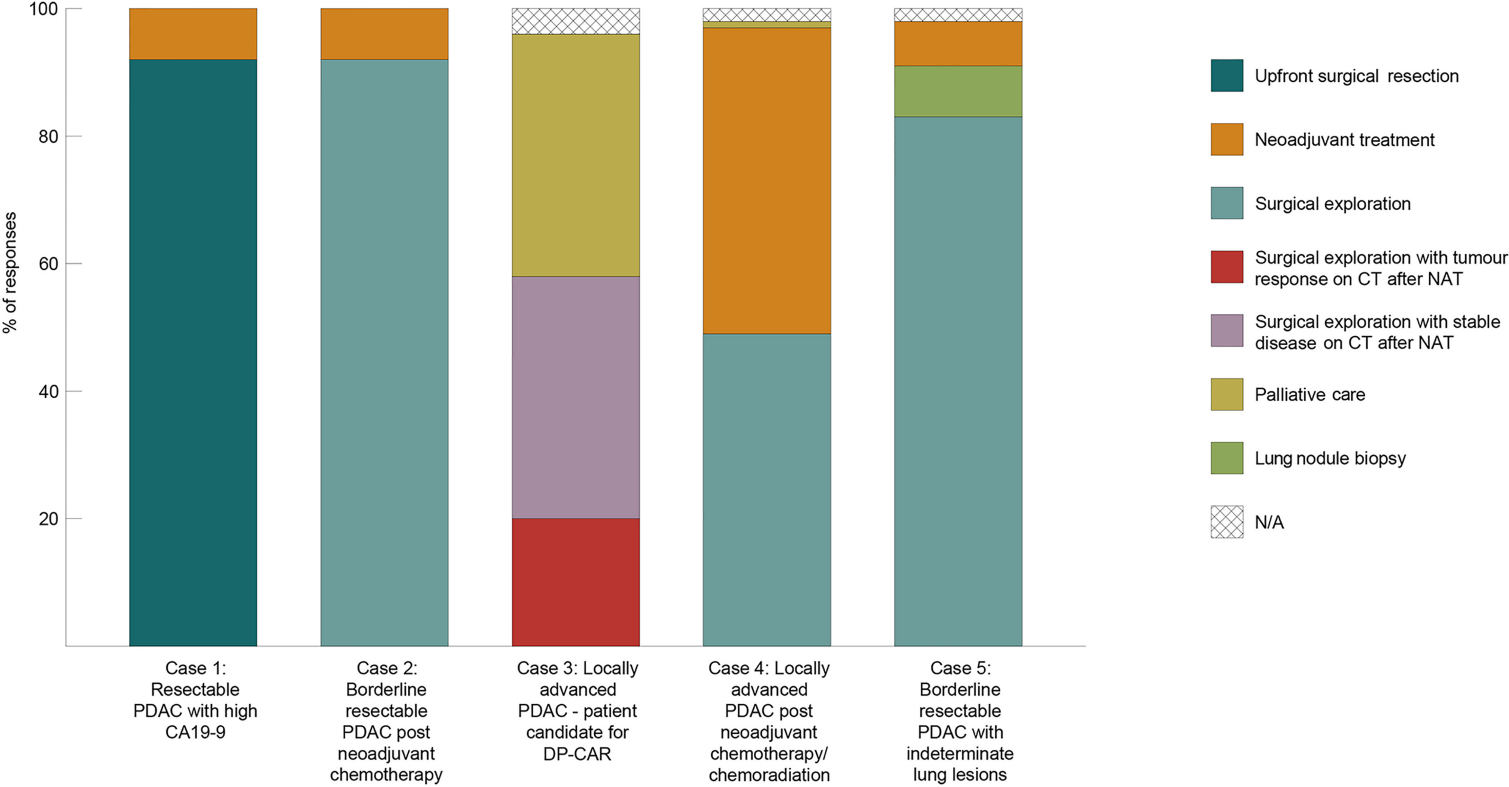
Allocation of responses regarding PDAC surgical management in five different clinical scenarios.

The response distribution for the clinical vignettes is available in **Figure 2**. The majority of surgeons would offer surgical resection in a patient with resectable PDAC and high CA19-9, and surgical exploration in a patient with BR-PDAC and stable disease on CT after four months of gemcitabine/nab-paclitaxel (n=60, in both cases). In the latter case, the rationale for continuation of NAT (n=5) and re-evaluation was mainly concern about R0 margin resection and uncertainty regarding tumour biology; all surgeons who advocated for NAT practised in high or very high-volume institutions and most preferred additional systemic treatment with FOLFIRINOX (n=3).

The third clinical vignette presented a fit patient with a pancreatic body PDAC and involvement of the coeliac artery, who is a potential candidate for distal pancreatectomy with coeliac artery resection (DP-CAR). Most participants opted for NAT and surgical exploration after stable disease/no progression or treatment response on CT per RESIST 1.1 criteria (20) (38% and 20%, respectively). Interestingly, 38% of participants (n=25, similarly distributed between the three tiers) replied that they would refer the patient for palliative systemic treatment without an option for surgical exploration. In a similar scenario of a young patient with LA-PDAC, half of surgeons (n=32) would offer surgical exploration after approximately one year of systemic treatment without disease progression; 78% of them (n=25) would declare the primary tumour unresectable if arterial reconstruction were deemed necessary intraoperatively. The final vignette described the likely scenario of a patient with a BR-PDAC and an indeterminate lung nodule on imaging: 15% of participants would not proceed with exploration after four months of NAT and would require biopsy of the lung lesion or continuation of systemic treatment.

### Intra-Institutional Variation

**Figure 3:**
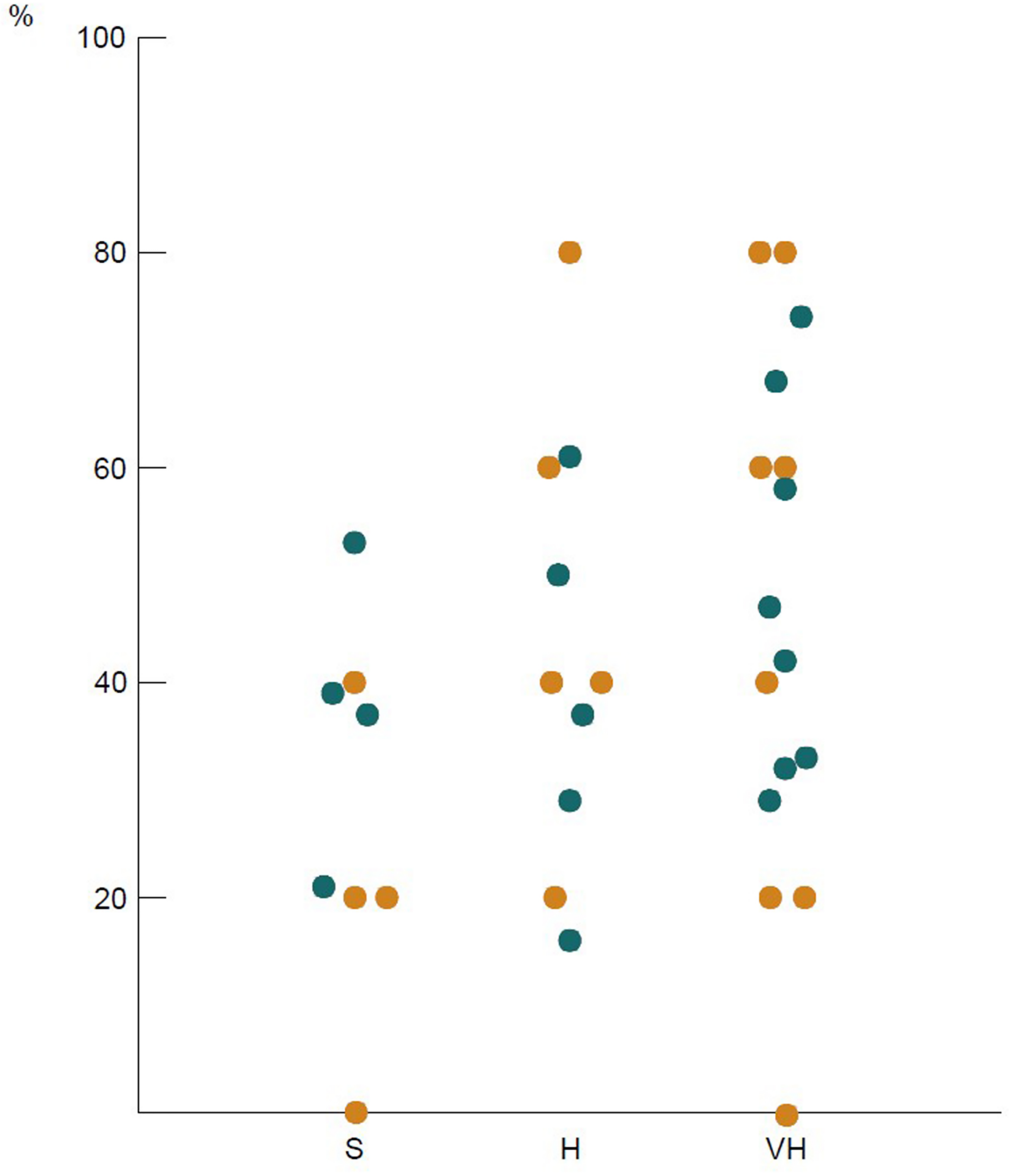
Intra-institutional variation in responses: each dot represents the percentage of questions across the survey with different answers from surgeons in the same institution. Columns refer to standard (S), high (H), and very high (VH) volume hospitals; orange dots: clinical vignettes, blue dots: other questions.

Review and analysis of responses from surgeons in the same institution demonstrated significant variation in practice (**Figure 3**). This was reflected in both the individual questions and the clinical vignettes, where 20-80% of responses were different within the same centre. Convergence in decision-making and surgical practice was not observed in any of the three tiers. Complete agreement in clinical vignette responses was observed only from surgeons in one institution in the standard volume and one in the very-high volume tier (25% and 12.5%, respectively).

## Discussion

The rationale for this study was to assess and define variations in surgical practice and to our knowledge is the first to provide an insight into the landscape of current surgical management of non-metastatic PDAC in the UK. The replies collected from this survey represent the majority of surgeons who perform pancreatic resections nationally and demonstrate significant variability in therapeutic approach and decision-making, both in inter-, and intra-institutional level. These differences were more prominent in the management of BR-PDAC and LA-PDAC, primarily in terms of anatomic criteria for resectability of the primary tumour.

The highest rate of agreement among the responders was observed in the management of patients with resectable PDAC; approximately 90% advocate for upfront surgery and adjuvant systemic treatment. Traditionally, surgical resection of primary pancreatic tumours without vessel involvement is the treatment of choice in eligible patients (21) and is recommended by national and international guidelines (22). Moreover, “fast-track” clinical pathways can facilitate prompt patient access to surgery and result in higher resection rates and improved outcomes (23). Although postoperative complications can delay the administration of systemic treatment (24, 25), recent advancements in chemotherapy (6, 7) have had a significant impact in recurrence-free and overall survival rates (26).

However in this survey, treatment of patients with resectable PDAC was not significantly affected by high CA19-9 levels or poor patient performance status (PS) (27). More specifically, 86% and 51% of surgeons would offer upfront resection in patients with CA19-9 >1000U/ml or PS ≥2, respectively. CA19-9 is currently the only available biomarker in clinical practice for PDAC and despite mediocre sensitivity and specificity, high values are suggestive of aggressive tumour biology (28) and have been associated with early disease recurrence (29). The study of biological behaviour in PDAC gradually introduced further criteria for resectability beyond anatomic considerations (11, 30) aiming at the improvement of recurrence rates and overall survival. Since optimal outcomes in PDAC are observed when a combination of local and systemic treatment is provided, the concept of NAT in resectable PDAC was developed with two objectives in mind: 1. test tumour biology and better select patients who are eligible for surgery, and 2. increase the percentage of patients who received chemotherapy. Retrospective studies on NAT in resectable PDAC have demonstrated increased rates in delivery of intended systemic therapy (31), and the recent SWOG S1505 trial showed that this is a feasible approach that needs to be investigated further (32). In this study, less than 10% of participants from high and very high-volume institutions would routinely offer this approach and only within a clinical trial, as suggested by the NICE guidelines.

Limited variation was identified in the management of BR-PDAC, since the majority of surgeons support utilisation of NAT and surgical exploration. The concept of preoperative chemotherapy and chemoradiation in BR-PDAC was developed under the prism of vessel involvement by the tumour and increased probability of R1 resection with a subsequent effect on patient survival (4, 33). Even though the percentage of patients who eventually undergo surgical resection may be lower, the recent prospective PREOPANC and ESPAC-5F trials demonstrated improved tumour response and a survival benefit with NAT and surgery (12, 13), indicating a paradigm shift in the surgical management of these patients.

Advancements in surgical techniques, perioperative care, and systemic treatment options have significantly improved outcomes in patients with LA-PDAC. Recent retrospective series have demonstrated that surgical resection in LA-PDAC post induction therapy is feasible (34–36) and has a direct impact on patient survival (37, 38). However, most of the current practice is based on observational data and therefore, the lack of consensus in LA-PDAC management shown in this study is not surprising. Participants expressed different opinions regarding time and regimen of induction therapy, most likely based on single-centre retrospective data. These results coincide with differing views regarding number of chemotherapy cycles, or any survival benefit in utilisation of radiation therapy in the preoperative setting (39, 40).

Since tumour response to induction therapy in LA-PDAC is evaluated almost exclusively based on imaging, anatomic criteria are primarily utilised by surgeons to assess resectability and reconstructability. This combination of subjective radiological evaluation and low specificity and sensitivity of imaging modalities (41) can explain the limited role of surgery in LA-PDAC demonstrated in this study. The role of CT/PET has been previously investigated regarding assessment of resectability and has proven to provide an advantage in identifying active disease versus fibrosis, especially after neoadjuvant treatment (42). Another important factor appears to be the limited exposure to complex vascular reconstruction: participants from standard-volume hospitals performed significantly smaller number of vein resections and subsequently were more likely to declare a locally advanced tumour as unresectable, and vice versa. However, almost all surgeons in this study would declare a tumour unresectable intraoperatively if arterial involvement were identified, particularly of the superior mesenteric artery. Arterial resection and reconstruction in PDAC have been previously reported in very selective cases (43), but is characterised by high rates of postoperative complications and questionable survival benefit (44).

The variability in the assessment of patients with PDAC shown in this study indicates that the decision-making process is a combination of guideline utilisation, surgical skills, and individual experience within an MDT setting. Previous studies have reported differences in MDT outcomes between institutions and deviations in assessment of resectability or tumour response (45–47), extending beyond classification systems. This study further underlines that these differences are essentially present on an intra-institutional level and illustrates the necessity for standardisation of MDT reporting, which is also supported by the majority of participants.

This study has several important limitations. Firstly, even though response rate was >50%, the sample size was relatively small (n=65) making identification of statistically significant differences problematic. Additionally, the survey was eponymous and therefore research bias that may have affected the responses (Hawthorne effect) was introduced. Limited information regarding previous exposure of participating surgeons to the management of BR-DPAC and LA-PDAC post neoadjuvant therapy were also available. Most importantly, it remains unclear if the demonstrated differences in management translate into differences in patient outcomes, since they were not the scope of this study. Lastly, this study was primarily performed before the ongoing COVID-19 pandemic, which has significantly affected the management of PDAC patients and is expected to have significant effect on resection rates and survival (48, 49).

Following previous reports (15), the present study shows wide variations in the surgical management of non-metastatic PDAC across the UK. Even though pancreatic surgery is centralised in high-volume centres, a need for creation of a nationwide patient outcome database is underlined. This will allow identification and standardisation of optimal clinical pathways *via* audit processes within the NHS, similar to the National Surgical Quality Improvement Program in the United States and the British Transplantation Society in the United Kingdom, aiming primarily at the complex management of patients with BR and LA-PDAC.

## Data Availability Statement

The raw data supporting the conclusions of this article will be made available by the authors, without undue reservation.

## Author Contributions

All authors have contributed equally to this manuscript in terms of data acquisition and analysis, and manuscript preparation and refinement.

## Conflict of Interest

The authors declare that the research was conducted in the absence of any commercial or financial relationships that could be construed as a potential conflict of interest.

## Publisher’s Note

All claims expressed in this article are solely those of the authors and do not necessarily represent those of their affiliated organizations, or those of the publisher, the editors and the reviewers. Any product that may be evaluated in this article, or claim that may be made by its manufacturer, is not guaranteed or endorsed by the publisher.
