# Supplementary figures and images for: Surgical Management of Non-Metastatic Pancreatic Cancer in the United Kingdom: Results of a Nationwide Survey on Current Practice
Source: Front Oncol. 2021 Dec 23;11:791946. doi: 10.3389/fonc.2021.791946 (PMC8733562; doi:10.3389/fonc.2021.791946)

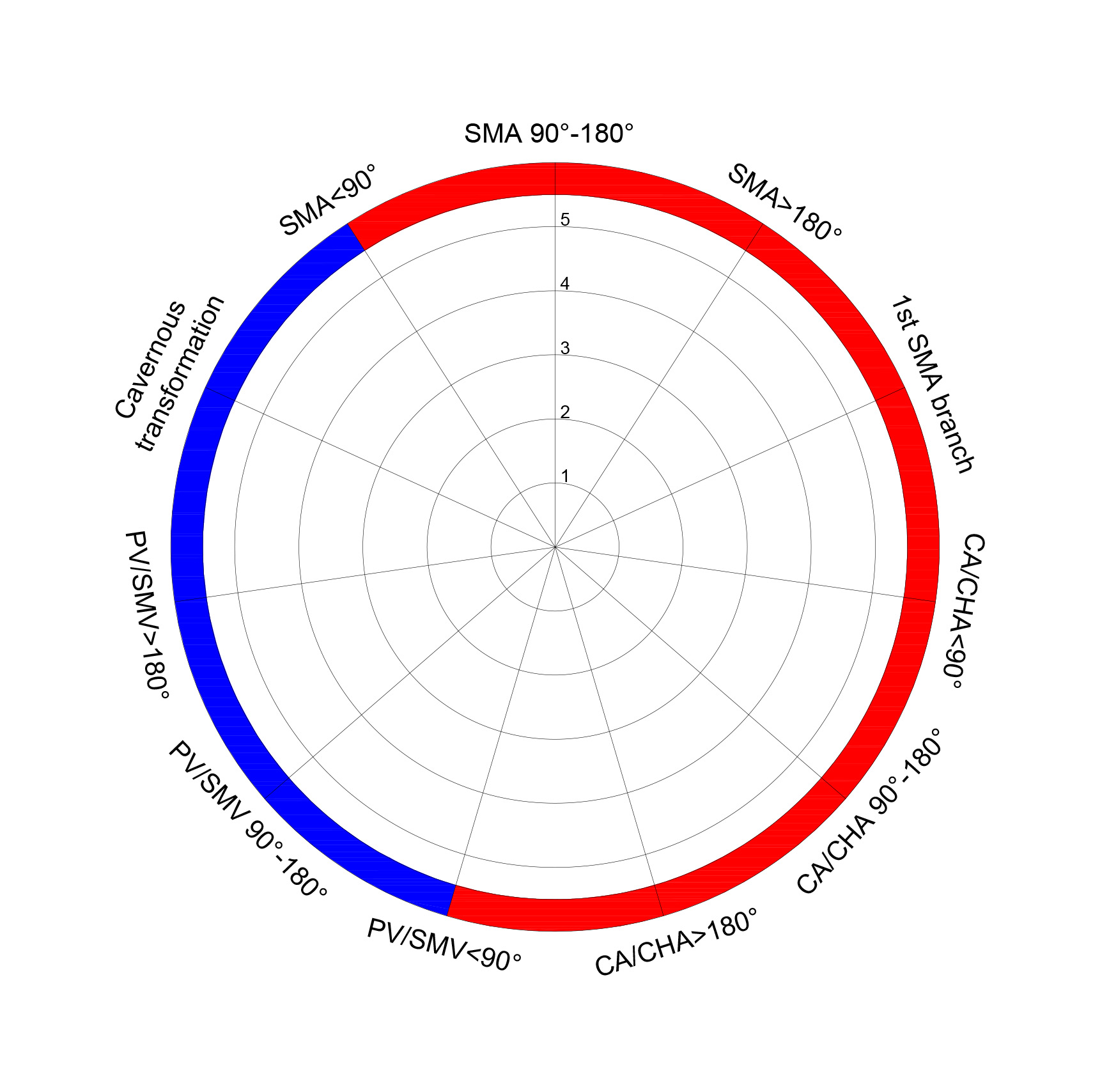

Supplement: Supplementary file 1 [file Image_1.jpeg]
