# Supplementary material for: Surgical Management of Non-Metastatic Pancreatic Cancer in the United Kingdom: Results of a Nationwide Survey on Current Practice
Source: Front Oncol. 2021 Dec 23;11:791946. doi: 10.3389/fonc.2021.791946 (PMC8733562; doi:10.3389/fonc.2021.791946)
Supplement: Supplementary file 2 [file DataSheet_1.pdf]

Current UK practice in the clinical management of Non-metastatic pancreatic cancer

You can find out what we do with your data in the Birmingham Surgical Trials Consortium (BiSTC) REDCap privacy notice.

Re: Roux/AUGIS (RCS Eng) collaborative study - Current UK practice in the clinical management of Non-metastatic pancreatic cancer

Dear colleague,

The RICOCHET audit has established there is considerable national variation in the management of pancreatic cancer. In addition, at the PSGBI Borderline/Locally Advanced Pancreatic Cancer meeting in November 2018 the body of surgeons and physicians agreed that work in this area was imperative, to standardise definitions and practice. As a first step, we plan to establish the current opinion for clinical decision making in the management of non-metastatic pancreatic cancer among all UK pancreatic centres via a survey.

The survey is being conducted by the Roux Group (the new AUGIS Trainees group), in association with AUGIS/RCS, and we would be very grateful for your contribution to ensure opinions from all pancreatic centres in the UK are represented. The survey consists of 16 questions and 5 case vignettes, and should take no longer than 10 minutes to complete. Surgeon name and centre name are used for the purpose of analysis only and to prevent duplication of data. The analysis of this data will be anonymised and not include identifiable surgeon or centre information.

The results from this survey will help to inform future studies in this challenging area.

Thank you for your assistance,

Kind regards

What is your name?

____
((forename, surname))

What is your email address?

____

Are you happy to be contacted about future studies in pancreatic cancer?

- ◯ Yes
- ◯ No

Neo-Adjuvant Chemotherapy

Do you use Neoadjuvant treatment in Resectable (i.e. no vascular involvement) Pancreatic Cancer?

- ☐ Routinely
- ☐ Selectively (please define next)
- ☐ Never

Please define how you selectively use neoadjuvant treatment in resectable pancreatic cancer

____

In patients who are PS 0-1, which neoadjuvant chemotherapy regimen do most of your patients receive?

- ☐ FOLFIRINOX
- ☐ Gemcitabine +/-capecitabine
- ☐ Gemcitabine/nab-paclitaxel
- ☐ Routinely use radiotherapy with NAT?
- ☐ Routinely use IORT with NAT?
- ☐ Routinely use IRE with NAT?

For frail and elderly patients who are potentially resectable do you give NAT?

- ◯ Yes
- ◯ No

For frail and elderly patients who are potentially resectable that you give NAT to, which regimen(s) do you use?

- ☐ FOLFIRINOX
- ☐ Gemcitabine +/-capecitabine
- ☐ Gemcitabine/nab-paclitaxel

Resectability

Would you resect a patient with the following adverse features?

- ☐ raised CA19-9 (in the absence of metastatic disease on full staging)
- ☐ PS 2
- ☐ PS 3

Please specify cut off for CA 19.9 for operability

- ◯ 0-250
- ◯ 250-500
- ◯ 500-1000
- ◯ 1000+

What staging system is your MDT using to classify tumours/resectability (select multple if appropriate)

- ☐ NCCN
- ☐ Alliance
- ☐ MD Anderson
- ☐ Other (state next)
- ☐ None

Please state 'other' staging system used to classify tumour/resectability

____

In general, how useful do you find current staging systems?

- ◯ Very useful
- ◯ Useful
- ◯ Not very useful
- ◯ Useless

Approximately how many vascular resections have you be involved in (either primary operating surgeon or assistant) in the past 2 years?

- ◯ 0
- ◯ 1
- ◯ 2-5
- ◯ 6-10,
- ◯ >10
- ◯ Don't know

What types of interposition graft do you use?

- ☐ Cadaveric
- ☐ Autologous left renal
- ☐ Autologous saphenous
- ☐ Autologous jugular
- ☐ Prosthetic
- ☐ Bovine patch
- ☐ N/A

Which of the following techniques do you perform for vein resection?

- ☐ Wedge/side-bite
- ☐ End to end
- ☐ Interposition graft
- ☐ N/A

In general, how do you manage venous involvement in pancreatic cancer (assume patient is fit for surgery)?

- ☐ Upfront surgery
- ☐ NAT
- ☐ Unresectable/palliative

Would the following scenario change this treatment plan (select those only where treatment is different from above)?

- ☐ PV and/or SMV - minimal contact (< 90 degrees)
- ☐ PV and/or SMV - moderate contact (90-180 degrees)
- ☐ PV and/or SMV - major contact (>180 but < 360 degrees)
- ☐ PV and/or SMV - complete contact (360 degrees)
- ☐ PV and/or SMV - complete occlusion and varices
- ☐ Short segment involvement unlikely to need graft
- ☐ Longer segment involvement likely to need graft
- ☐ Involvement of first jejunal branch

Please rate the importance of the following when you consider venous involvement:

	Very important	Important	Mildly important	Not important
Length of involvement	○	○	○	○
Amount of circumferential contact	○	○	○	○
Degree of narrowing	○	○	○	○
Presence of varices with occluded vein	○	○	○	○
Involvement of first jejunal branch	○	○	○	○

In general, how do you manage arterial involvement in pancreatic cancer (assume patient is fit for surgery):

- ○ Upfront surgery
- ○ NAT
- ○ Unresectable/palliative

Would the following scenario change your treatment plan (re: arterial involvment)?

	Upfront resectable	Neo-adjuvant therapy	Unresectable/palliative
Minimal contact (< 90 degrees) with the SMA	○	○	○
Moderate contact (90-180 degrees) with the SMA	○	○	○
Major contact (>180 degrees but < 360 degree) with the SMA	○	○	○
Complete contact (360 degrees) with the SMA	○	○	○
Minimal contact (< 90 degrees) with Coeliac/CHA (but GDA clear and hepatic arteries clear)	○	○	○
Moderate contact (90-180 degrees) with Coeliac/CHA (but GDA clear and hepatic arteries clear)?	○	○	○

Major contact (>180 but < 360 degrees) with Coeliac/CHA (but GDA clear and hepatic arteries clear)	○	○	○
Complete contact (360 degrees) with Coeliac/CHA (but GDA clear and hepatic arteries clear)	○	○	○
Any contact with the common hepatic artery (CHA) including RHA-LHA bifurcation for pancreatic head tumours	○	○	○
Involving the 1st jejunal SMA branch	○	○	○

How important are the following factors when considering arterial involvement?

	Very important	Important	Mildly important	Not important
Length	○	○	○	○
Circumferential contact	○	○	○	○
Degree of narrowing	○	○	○	○
Which artery is involved i.e. SMA vs Coeliac	○	○	○	○

MDT Reporting

Is your MDT report:

- ○ Free text description
- ○ Template of options

	Definitely not	May be	Yes	Definitely
Would you like to use an MDT template to standardise reporting of disease and treatment?	○	○	○	○

Case 1

A 54-year old man presents with obstructive jaundice and a 24mm lesion in the pancreatic head. There is no vascular involvement and furter staging was negative for distant metastases; CA 19.9 was 1027 U/ml. The patient's performance status is 1 and BMI is 29.3.

Would you offer this patient an upfront resection?

- ○ Yes
- ○ No

If not, what alternative treatment/approach would you offer this patient?

- ○ CBD stent placement, neoadjuvant chemotherapy (FOLFIRINOX) and restaging
- ○ CBD stent placement, neoadjuvant chemotherapy (Gemcitabine based chemotherapy) and restaging
- ○ Neoadjuvant chemotherapy within a clinical trial

Case 2

A 69-year old lady with a 3cm pancreatic head adenocarcinoma and segmental encasement (involving >180 degrees) and narrowing of the portal/superior mesenteric vein confluence over 3cm, with no arterial involvement on initial staging. CA 19.9 at the time of diagnosis is 259 U/ml. She subsequently completed 4 months of gemcitabine/nab-paclitaxel. She now presents to your clinic with a repeat CA 19.9 of 51 U/ml. Restaging imaging showed no evidence of local progression or metastatic disease.

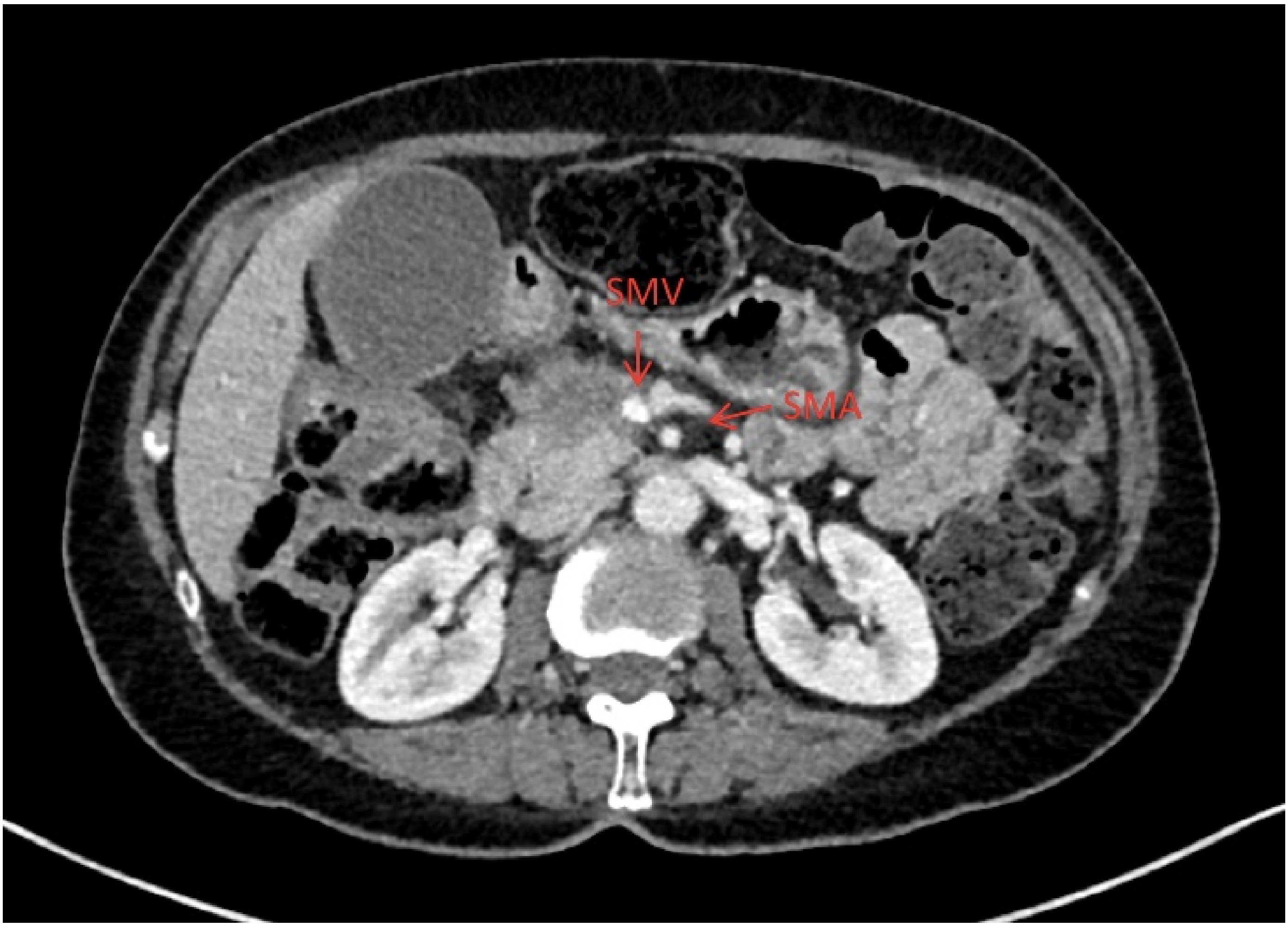

Would you offer this patient surgical exploration?	◯ Yes<br>◯ No
If not, what is your rationale?	◯ R0 resection concerns regarding the vein margin<br>◯ R0 resection concerns regarding the artery margin<br>◯ Complexity of potential reconstruction<br>◯ Uncertainty regarding treatment response/tumour biology
What alternative treatment/approach would you offer this patient?	◯ Additional chemotherapy (Gemcitabine/Nab-Paclitaxel)<br>◯ Additional chemotherapy (FOLFIRINOX)<br>◯ Stereotactic body radiation therapy and exploration in 6 weeks<br>◯ Conventional radiotherapy and maintenance Gemcitabine/Capecitabine for a month followed by surgical exploration<br>◯ Palliative chemotherapy

Case 3

A 62-year old ady presents with a 2 month history of epigastric discomfort, nausea, and unintentional weight loss of 5kg. Cross-sectional imaging demonstrated a 46mm pancreatic body lesion with encasement of the coeliac and common hepatic artery (arrow); there was no involvement of the gastroduodenal artery or the portal/superior mesenteric vein. The patient's performance status is 0 and CA 19.9 is 120 U/ml.

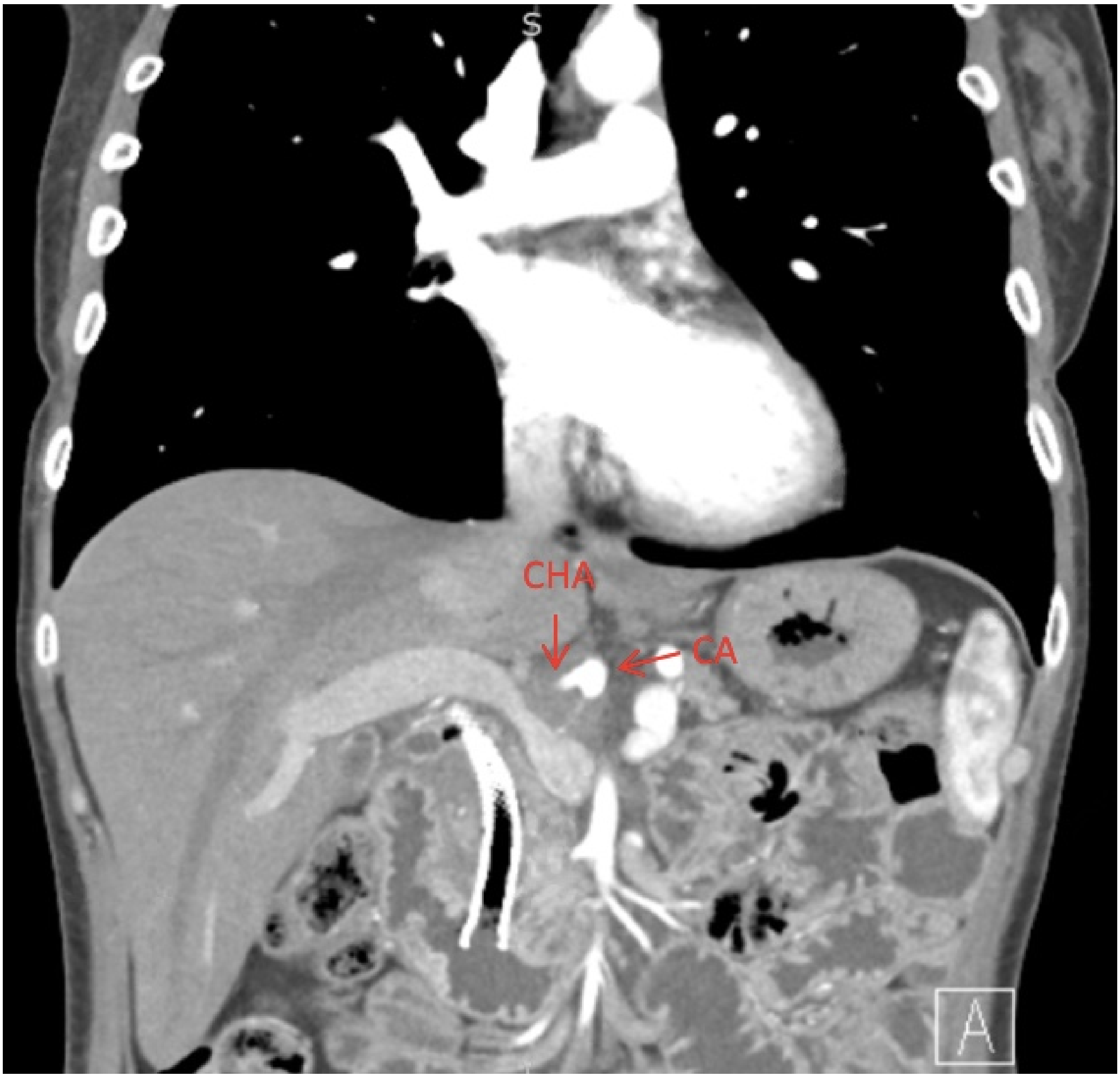

How would you proceed with the manangment of this patient?

- ◯ Upfront resection of the tumour (distal pancreatectomy with coeliac artery resection)
- ◯ Neoadjuvant chemotherapy and surgical exploration for distal pancreatectomy with coeliac artery resection only in case of treatment response per RECIST 1.1 i.e. radiologic improvement
- ◯ Neoadjuvant chemotherapy and surgical exploration for distal pancreatectomy with coeliac artery resection even in case of stable disease/non-progression per RECIST 1.1 i.e. stable on repeat CT
- ◯ Palliative chemotherapy

Case 4

A 37-year old male was diagnosed with a 37mm pancreatic adenocarcinoma of theuncinate process with 360 degree encasement of the superior mesenteric artery (2cm length, mesenteric vessels clear) and contact but no deformation of the superior mesenteric vein 6 months ago. Since then he underwent 6 months of neoadjuvant FOLFIRINOX. His latest CT demonstrates stable disease and a PET/CT shows no evidence of metastatic dissemination. The patient does not secrete CA 19.9 and all values have been less than 10.

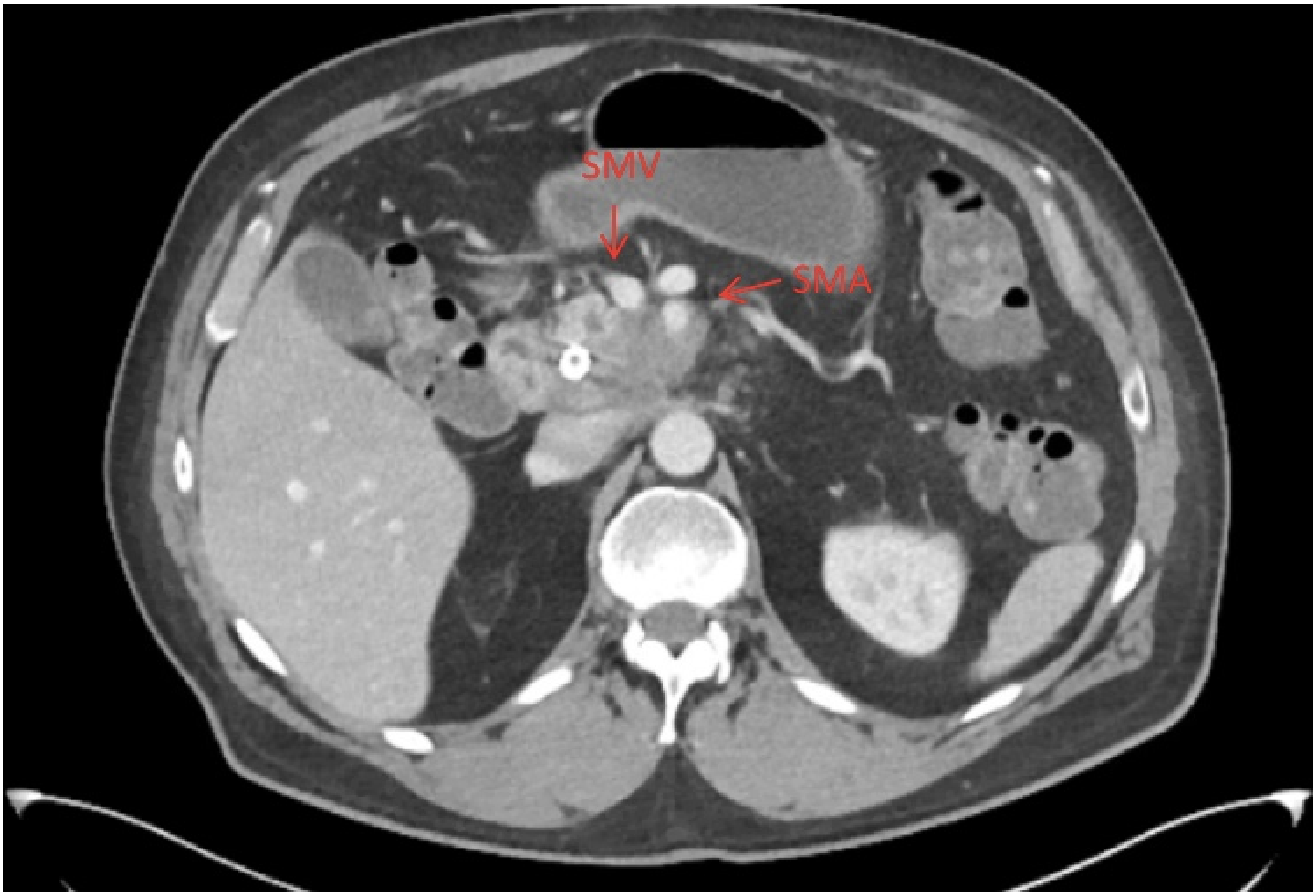

How would you manage this patient?	◯ Surgical exploration<br>◯ No surgical options/continue chemotherapy<br>◯ Best supportive care
When you proceed with surgical exploration, what intra-operative finding would make you declare the tumour unresectable?	☐ Need for arterial resection/reconstruction of the superior mesenteric artery<br>☐ Need for resection of the superior mesenteric vein and subsequent reconstruction with ligation of the splenic vein

Case 5

A 58-year old lady presents with a 29mm pancreatic adenocarcinoma of the pancreatic head, with less than 90 degrees contact of the portal vein and no arterial involvement. At staging, an indeterminate sub-centimetre lesion is identified in the lower lobe of the right lung. The patient undergoes 4 months of gemcitabine/abraxane, and repeat imaging demonstrates a decrease in the tumour size, now measuring 21mm without abutment of the vein and the same lung lesion. CA 19.9 was reduced from 1400 to 68 U/ml. The lung lesion showed no activity of PET/CT, but the report says metastasis cannot be excluded as PET activity in small lesions is unreliable.

How would you manage this patient?	◯ Surgical exploration and resection of the primary tumor followed by adjuvant chemotherapy<br>◯ Attempt to biopsy and characterise the pulmonary lesion<br>◯ Continue chemotherapy and restage in 2-4 months focusing on the pulmonary lesion<br>◯ Consider the patient to have stage IV disease and proceed with best supportive care

Unit practice

We would like to compare practice between and within units to understand variation further. Please complete th following details about your unit

Number of surgeons performing pancreatoduodenectomies at your unit?	__________________________________
In addition to pancreatic surgery do you perform pancreas and/or liver transplants?	◯ Yes<br>◯ No
What is the name of your centre?	__________________________________
